# Supplementary material for: A quieter state of charge and ultra-low-noise of the collective current in quasi-1D charge-density-wave nanowires
Source: Nat Commun. 2025 Dec 31;17:116. doi: 10.1038/s41467-025-67567-x (PMC12774952; doi:10.1038/s41467-025-67567-x)
Supplement: Supplementary file 1 — Supplementary Information [file 41467_2025_67567_MOESM1_ESM.pdf]

SUPPLEMENTAL INFORMATION

A Quieter State of Charge and Ultra-Low-Noise of the Collective Current in Quasi-1D Charge-Density-Wave Nanowires

Subhajit Ghosh[1,2], Nicholas Sesing[3], Zahra Ebrahim Nataj[1,2],Tina Salguero[3], Sergey Rumyantsev[4], Roger K. Lake[5], and Alexander A. Balandin[1,2,6×]

[1]Department of Materials Science and Engineering, University of California, Los Angeles, California, 90095 USA

[2]California NanoSystems Institute, University of California, Los Angeles, California, 90095 USA

[3]Department of Chemistry, University of Georgia, Athens, Georgia, 30602 USA

[4]Institute of High-Pressure Physics, Polish Academy of Sciences, Warsaw 01-142, Poland

[5]Department of Electrical and Computer Engineering, University of California, Riverside, California 92521, USA

[6]Center for Quantum Science and Engineering, University of California, Los Angeles, California, 90095 USA

[×] Corresponding author. E-mail: balandin@seas.ucla.edu

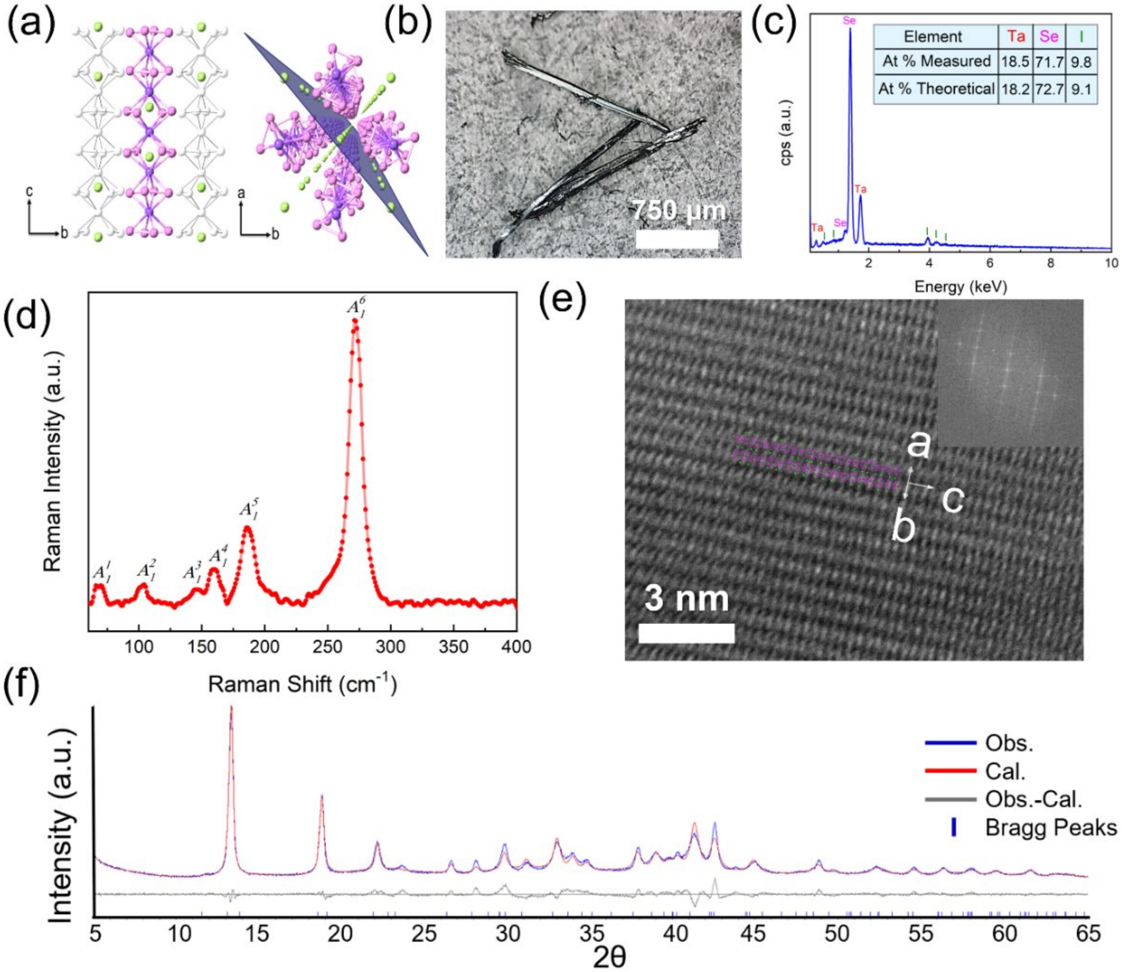

Supplemental Figure S1: **$(TaSe_4)_2I$ material characterizations**. (a) Left: $(TaSe_4)_2I$ atomic structure, shown down the a-axis, with a single $TaSe_4$ chain and iodide ions in color. Right: The atomic structure, shown down the c-axis, where the plane indicates the preferred (110) cleavage. (b) Photograph of CVT-grown $(TaSe_4)_2I$ crystals. (c) EDS spectrum from an exfoliated $(TaSe_4)_2I$ crystal, with peaks labeled with their corresponding elements. The inset table shows the atomic percentages of each element. (d) Raman spectrum of $(TaSe_4)_2I$ with labeled peaks. (e) HRTEM image of a $(TaSe_4)_2I$ nanowire with corresponding atomic structure overlay and inset FFT. (f) Rietveld refinement of the powder X-ray diffraction pattern of ground $(TaSe_4)_2I$ crystals.

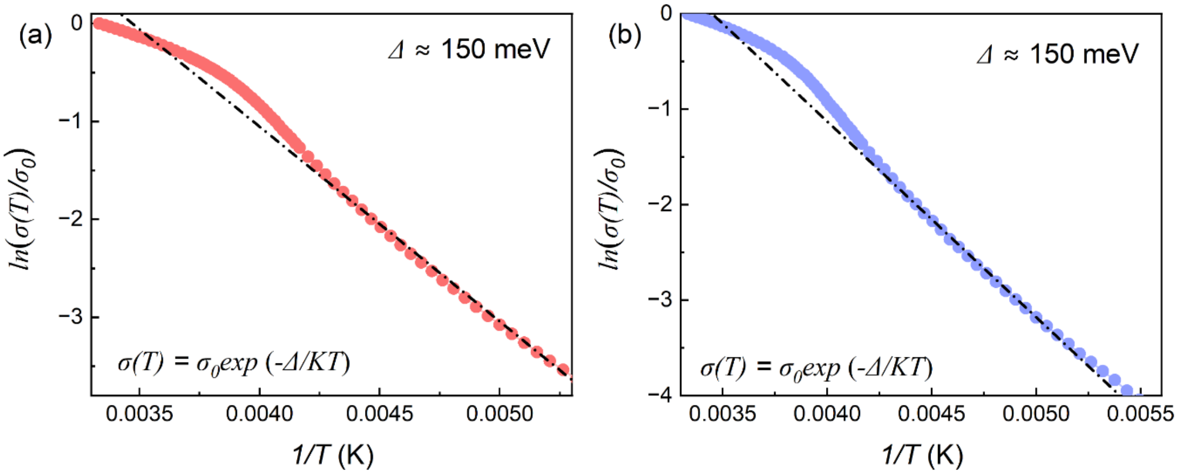

Supplemental Figure S2: Extraction of the CDW bandgap of $(TaSe_4)_2I$. The bandgap, E_g in the incommensurate CDW phase was extracted from the thermal activation equation $\sigma(T) = \sigma_0 \exp(-\Delta/K_B T)$, where σ_0 is the conductance at 300 K. The slope from the $\ln[\sigma(T)/\sigma_0]$ *vs.* $1/T$ plot provided the value of $-\Delta/K_B T$, where Δ is the activation energy, and K_B is the Boltzmann constant. The extracted bandgap, $E_g = 2\Delta$, is ~300 meV in both cases. Panels (a) and (b) show data for two different devices.

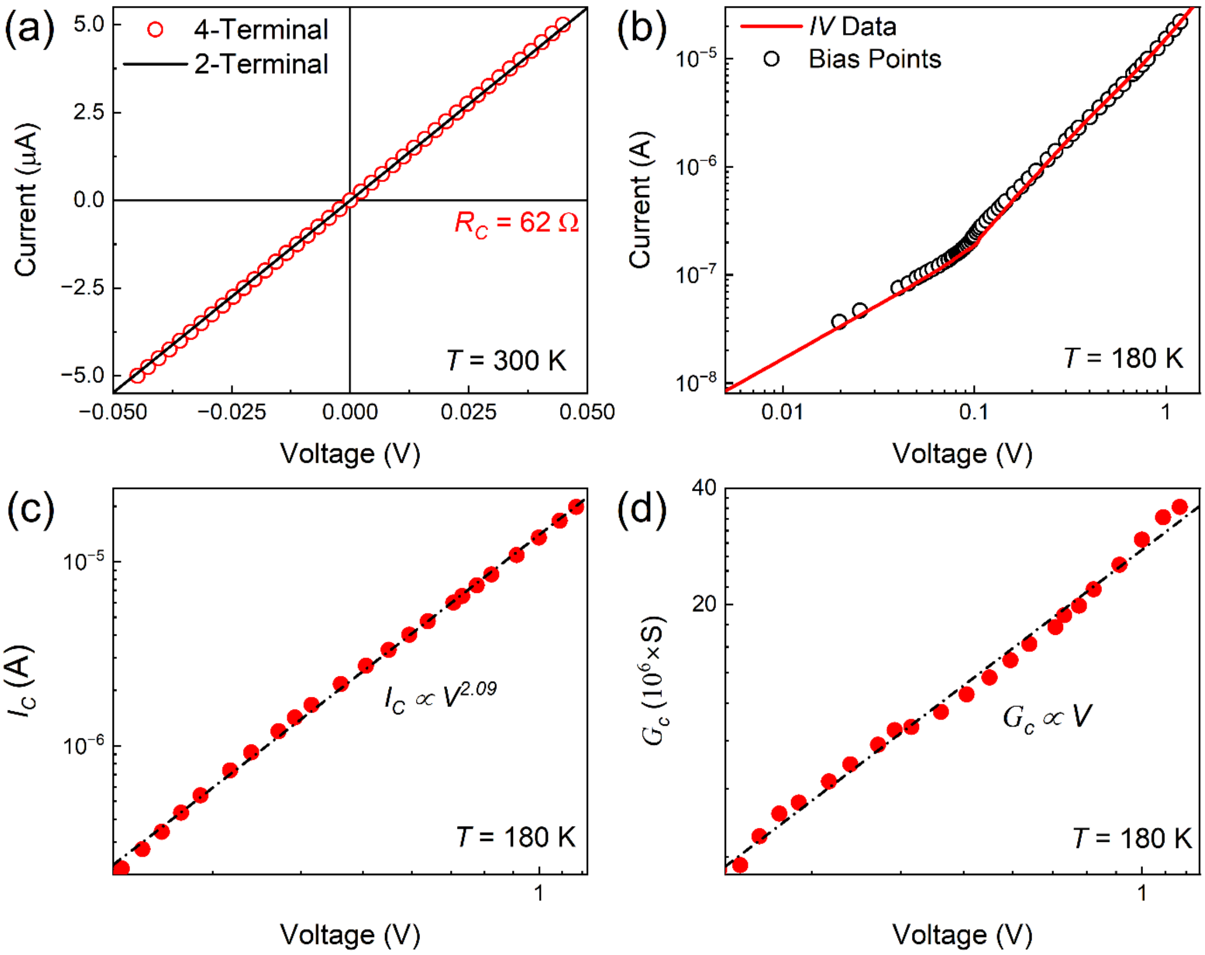

Supplemental Figure S3: **Additional transport data for a $(TaSe_4)_2I$ nanowire device**. (a) Room-temperature I-Vs were measured in the two-terminal and four-terminal configurations for the extraction of the contact resistance. The metal electrodes are Ohmic with low contact resistance, R_C, of less than 1% of the channel resistance. (b) The I-V characteristics for the 2-terminal devices and bias points in noise measurements plotted together. (c) CDW current, I_c, as a function of the applied bias, V, showing quadratic dependence. (d) CDW conductance, G_c, dependence on applied voltage, V.

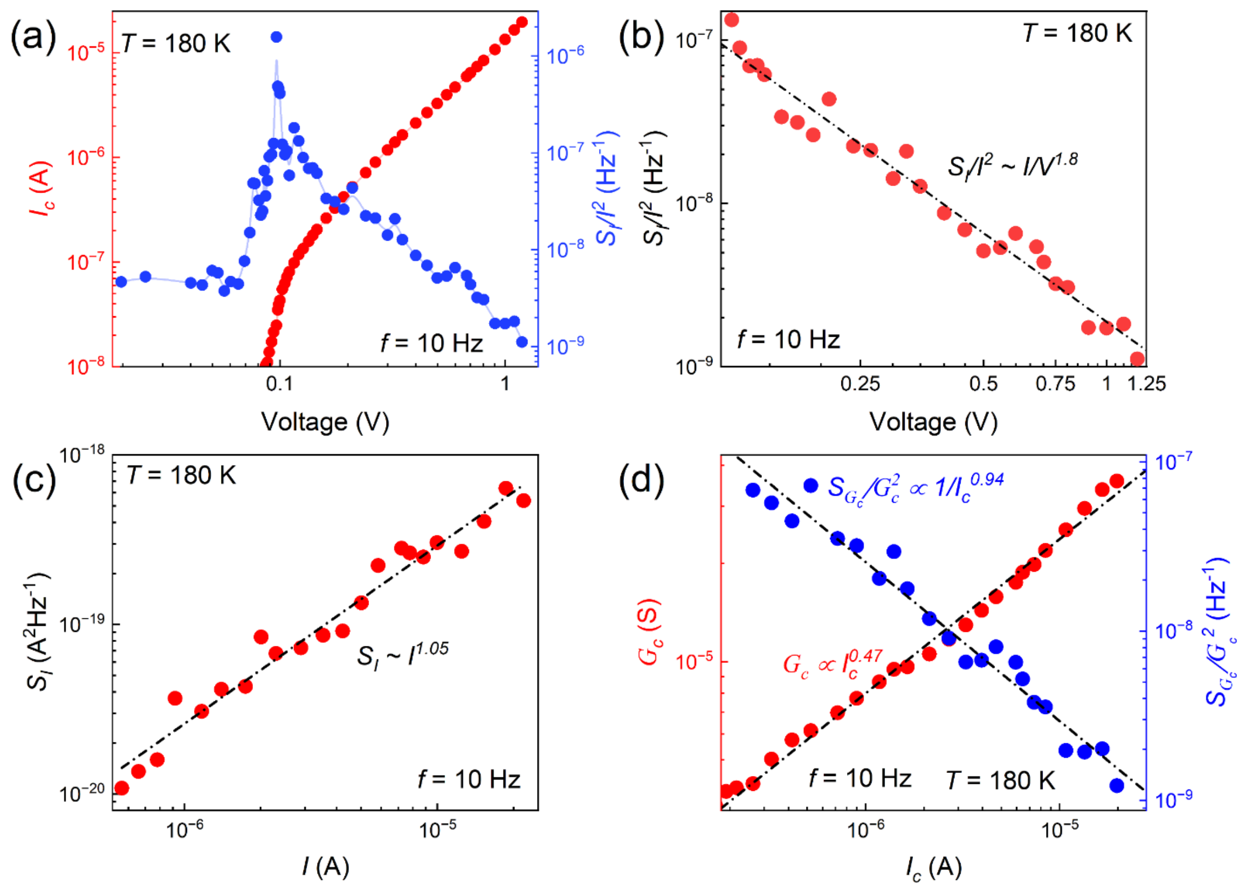

Supplemental Figure S4: Additional transport and noise data for a $(TaSe_4)_2I$ nanowire device. (a) CDW current, I_c, and normalized noise level, S_I/I^2, as a function of voltage, V, at T = 180 K. The noise peak appears near the depinning point as I_c starts increasing rapidly. (b) The current noise, S_I *vs.* V at f = 10 Hz. (c) The S_I *vs.* I reveals linear dependence. This dependence indicates that the normalized noise spectral density *reduces* with increasing current as $S_I/I^2 \propto 1/I$. (d) The G_c and $S_{G_c}/G_c{}^2$ dependence on I_c for comparison. The data are for device 1, described in the main text.

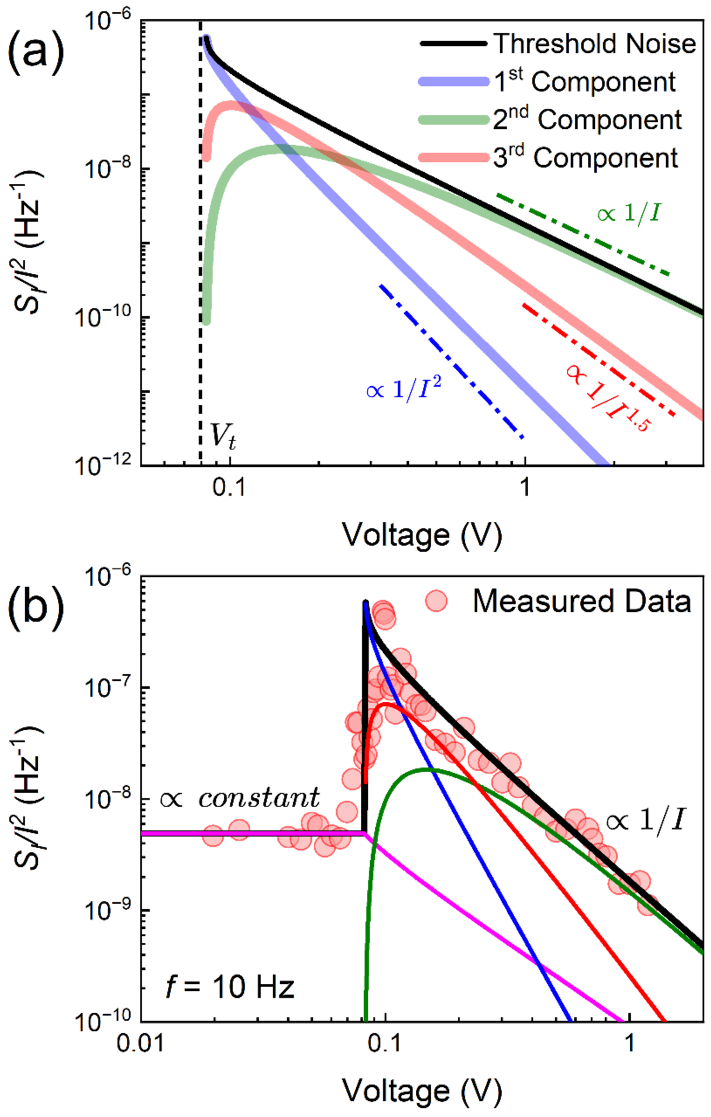

Supplemental Figure S5: **Theoretical analysis of noise data for $(TaSe_4)_2I$ nanowire devices**. (a) The threshold field fluctuations, $\langle \delta V_t^2 \rangle / V_t^2$, near V_t. The total "threshold" noise, $\langle \delta V_t^2 \rangle / V_t^2$, consists of three components that scale differently with bias. The blue line represents the first component, S_{t_1}/I^2, the green shows the second component, S_{t_2}/I^2, and the red line represents the third component, S_{t_3}/I^2. (b) All the noise components are superimposed on the experimental data. The magenta-colored line represents the weighted normal electron noise dependence on bias voltage. The blue, green, and red lines represent the first, second, and third components of the threshold field fluctuations, respectively. The black line shows the cumulative total noise behavior with the applied bias. The data are shown for device 1.

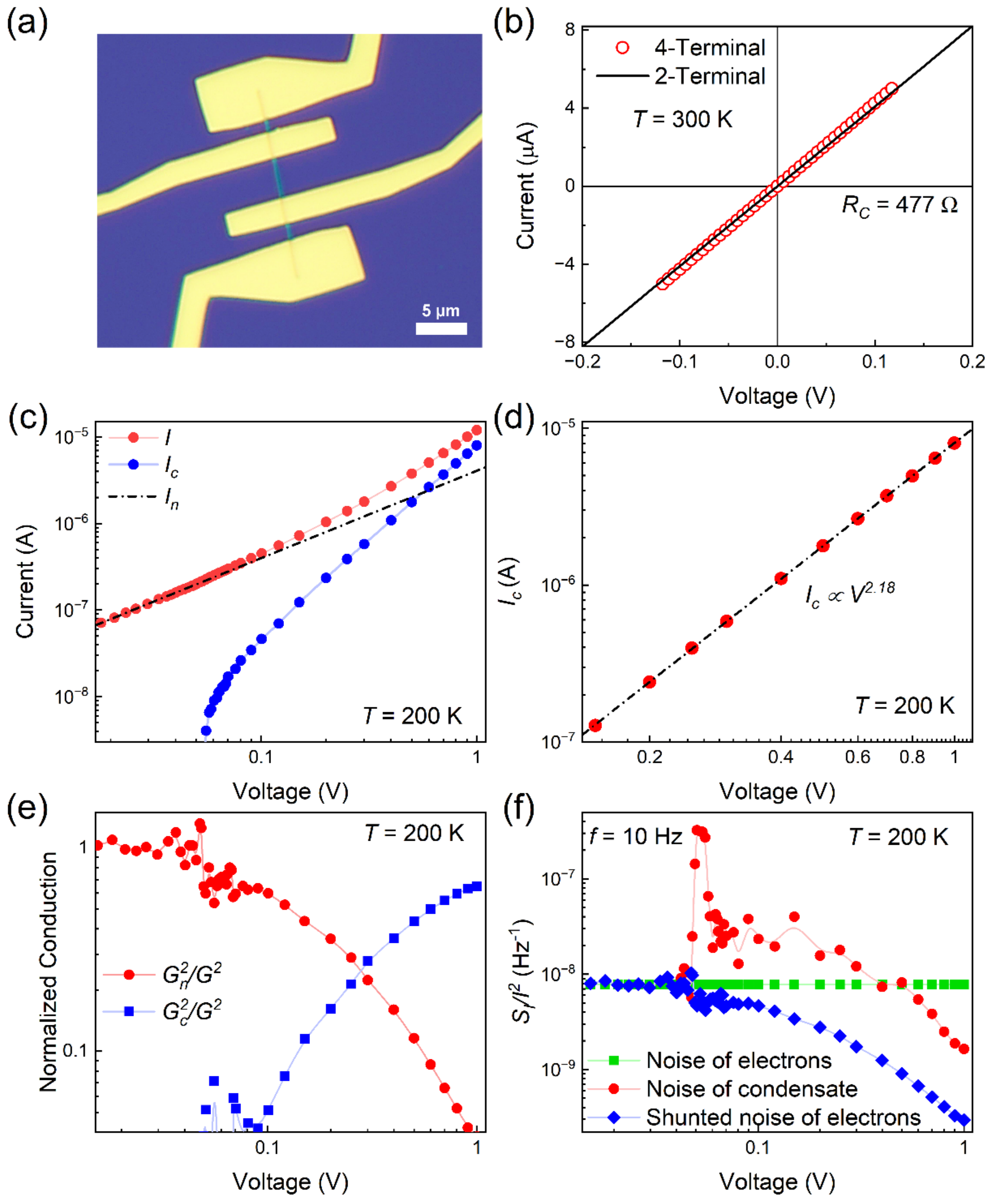

Supplemental Figure S6: **Transport and noise in a $(TaSe_4)_2I$ nanowire device.** (a) Optical microscopy image of device 2. (b) The two-terminal and four-terminal measured I-V characteristics of the middle channel with L =3.8 µm at T = 300 K. The contact resistance, R_c, is less than 2% of the intrinsic channel resistance. (c) The I-V characteristics at T = 200 K on a log scale, showing linear behavior below the threshold field, V_t, and super-linear thereafter. (d) The collective current dependence on bias, I_c vs. V. (e) The normalized conductance contribution of normal, $[G_n/G]^2$, and CDW currents, $[G_c/G]^2$, as a function of applied bias. (f) Noise of electrons (green symbols), weighted noise of electrons (blue), and weighted noise of the sliding CDW condensate (red) as a function of bias voltage. All data are for device 2.

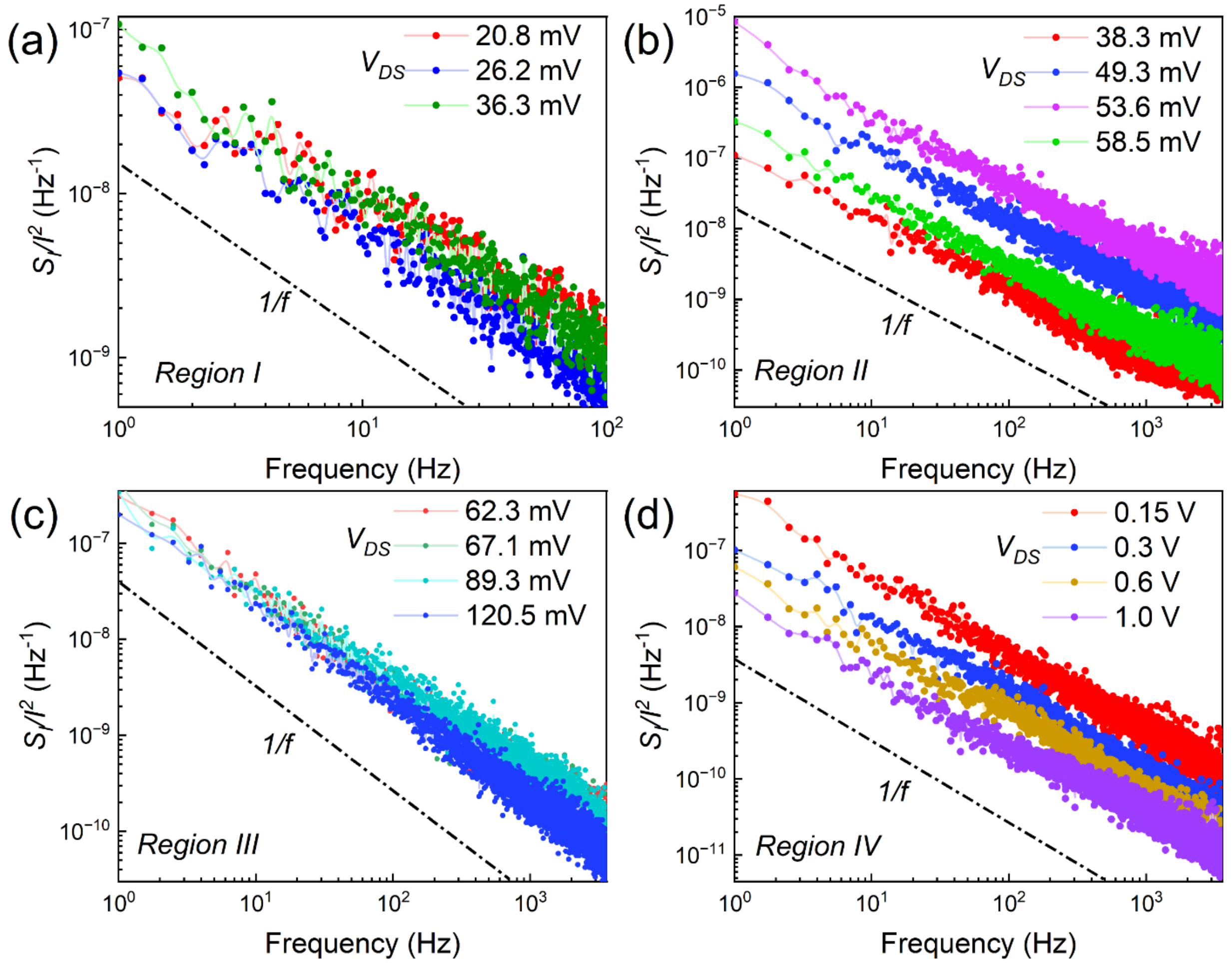

Supplemental Figure S7: **Noise spectra for a $(TaSe_4)_2I$ nanowire device at different bias ranges**. (a) The normalized noise spectral density, S_I/I^2, at (a) lower biases (region *I*), (b) across the depinning (region *II*), (c) beyond the depinning (region *III*), and (d) in the CDW sliding region (region *IV*). The noise is of *1/f* type, with some signatures of the Lorentzian bulges. The 60 Hz harmonics due to the power grid from the spectra were removed during data analysis. All data are for device 2.

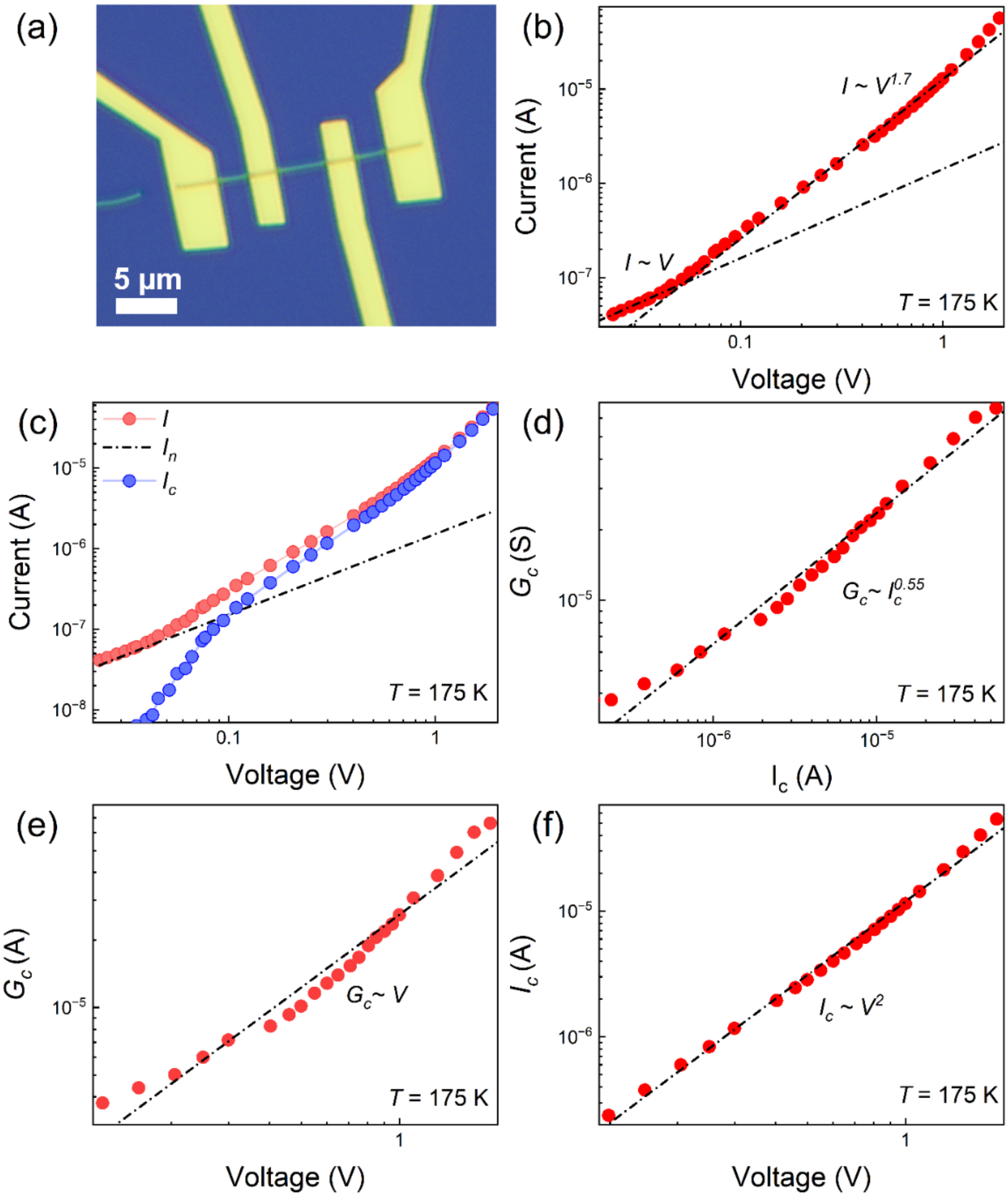

Supplemental Figure S8: **Transport data for a $(TaSe_4)_2I$ nanowire device**. (a) The optical microscopy image of the device. (b) The non-linear IV behavior at T = 175 K. (c) The linear current, I_n and collective current, I_c as extracted from the total current, I. (d) The CDW conductance, G_c, dependence on CDW current, I. (e) CDW conductance, G_c, linear dependence on applied voltage, V. (f) The CDW current, I quadratic dependence on voltage, V. All data are for device 3.

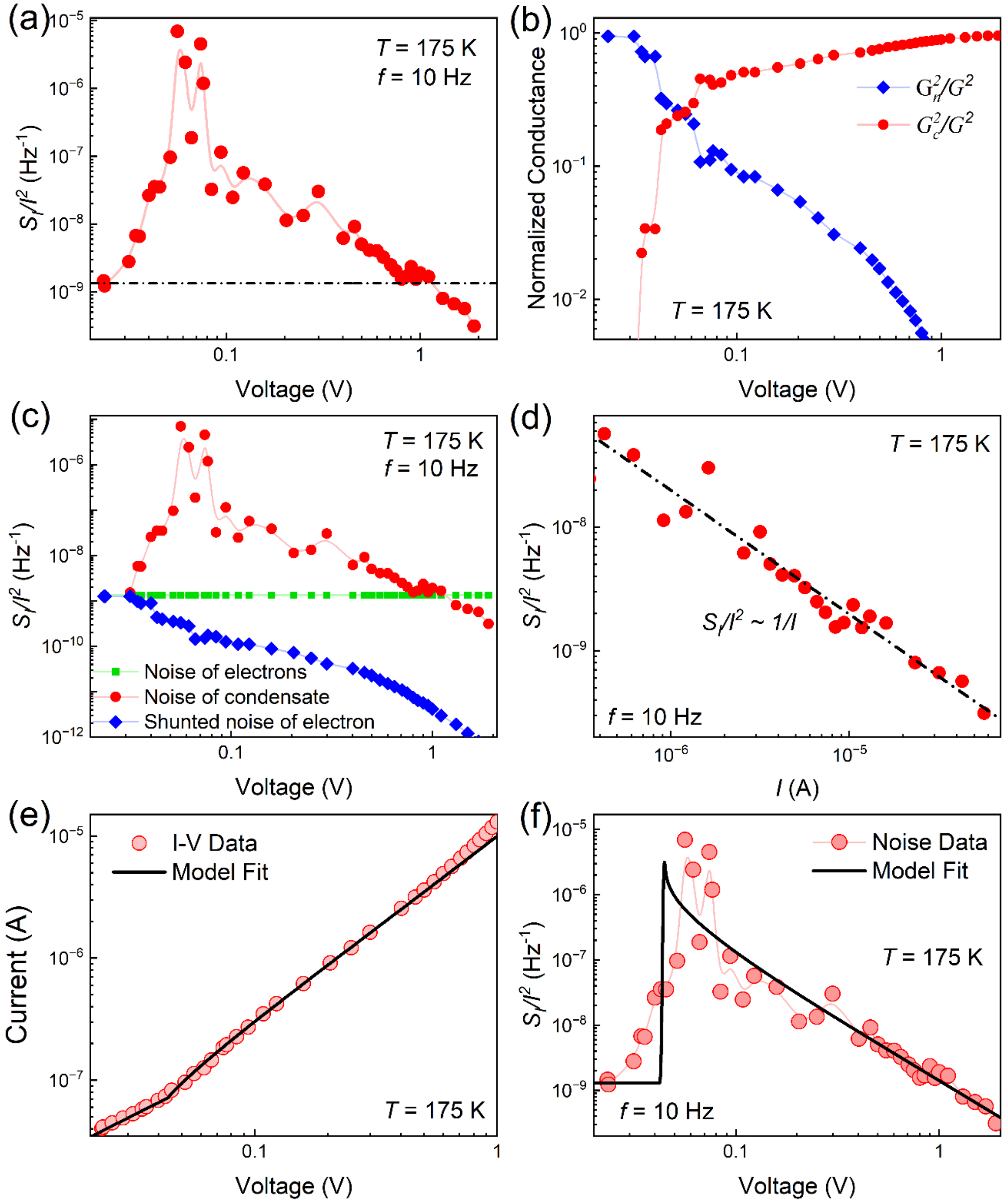

Supplemental Figure S9: **Noise data for a $(TaSe_4)_2I$ nanowire device**. (a) The noise, S_I/I^2, at fixed frequency f =10 Hz *vs.* bias voltage, measured at T = 175 K. The noise at higher biases goes below the linear noise. (b) The relative conductance contribution of normal, $[G_n/(G_n+G_c)]^2$, and CDW carriers, $[G_c/(G_n+G_c)]^2$, as a function of bias voltage. (c) Noise of electrons (green symbols), weighted noise of electrons (blue) and weighted noise of the sliding CDW condensate (red) as a function of bias voltage. (d) S_I/I^2, as a function of current scales inversely with the applied current. (e) The I-V data were fitted with the model equation. (f) The noise data fitted with the model. All data are for device 3.

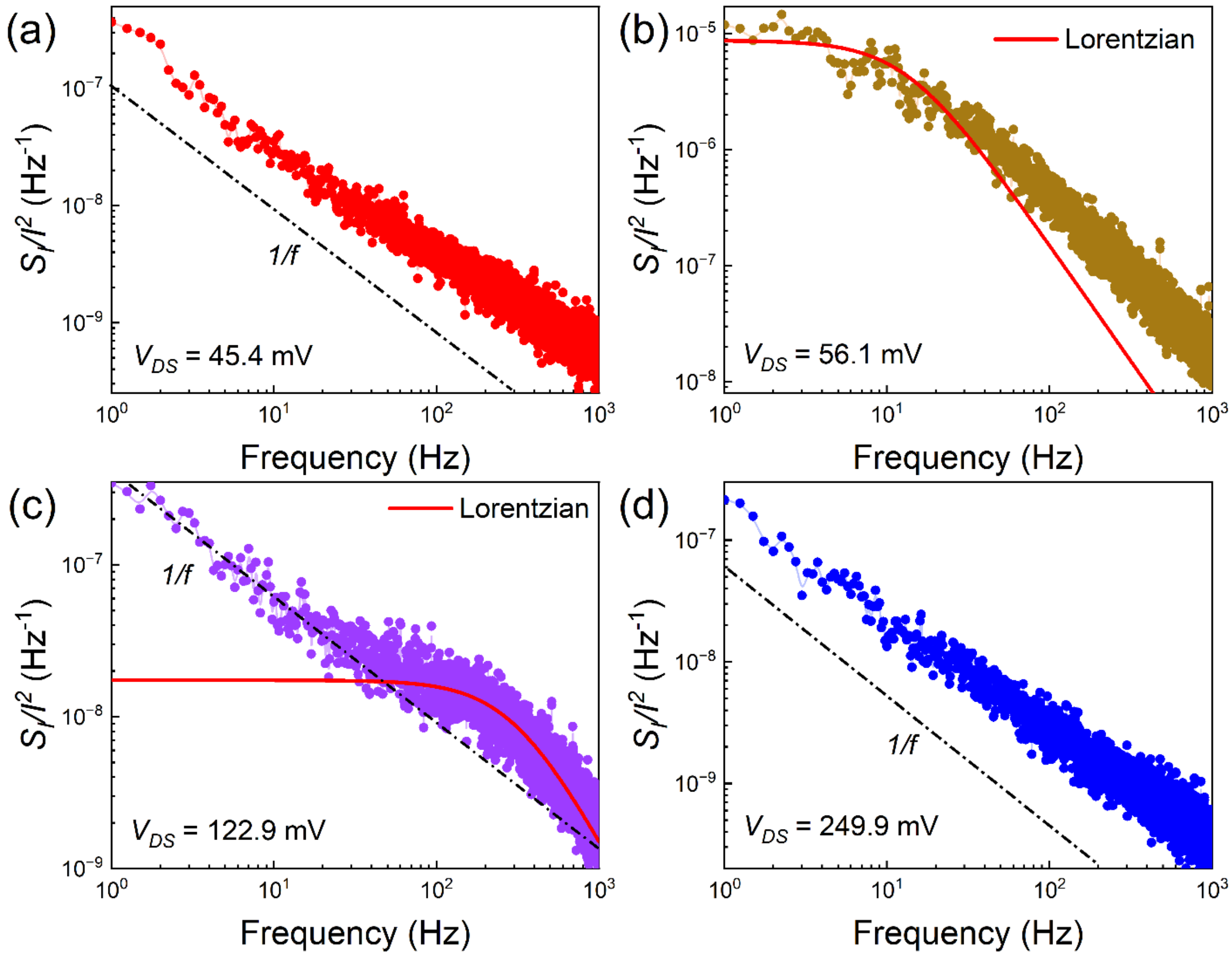

Supplemental Figure S10: **Noise spectra across the depinning for a $(TaSe_4)_2I$ nanowire device**. (a) The noise spectrum right before depinning is showing $1/f$ noise dependence. (b) and (c) show the emergence of Lorentzian across the depinning threshold. (d) The noise spectrum returns to $1/f$ type in the sliding regime. All data are for device 3.

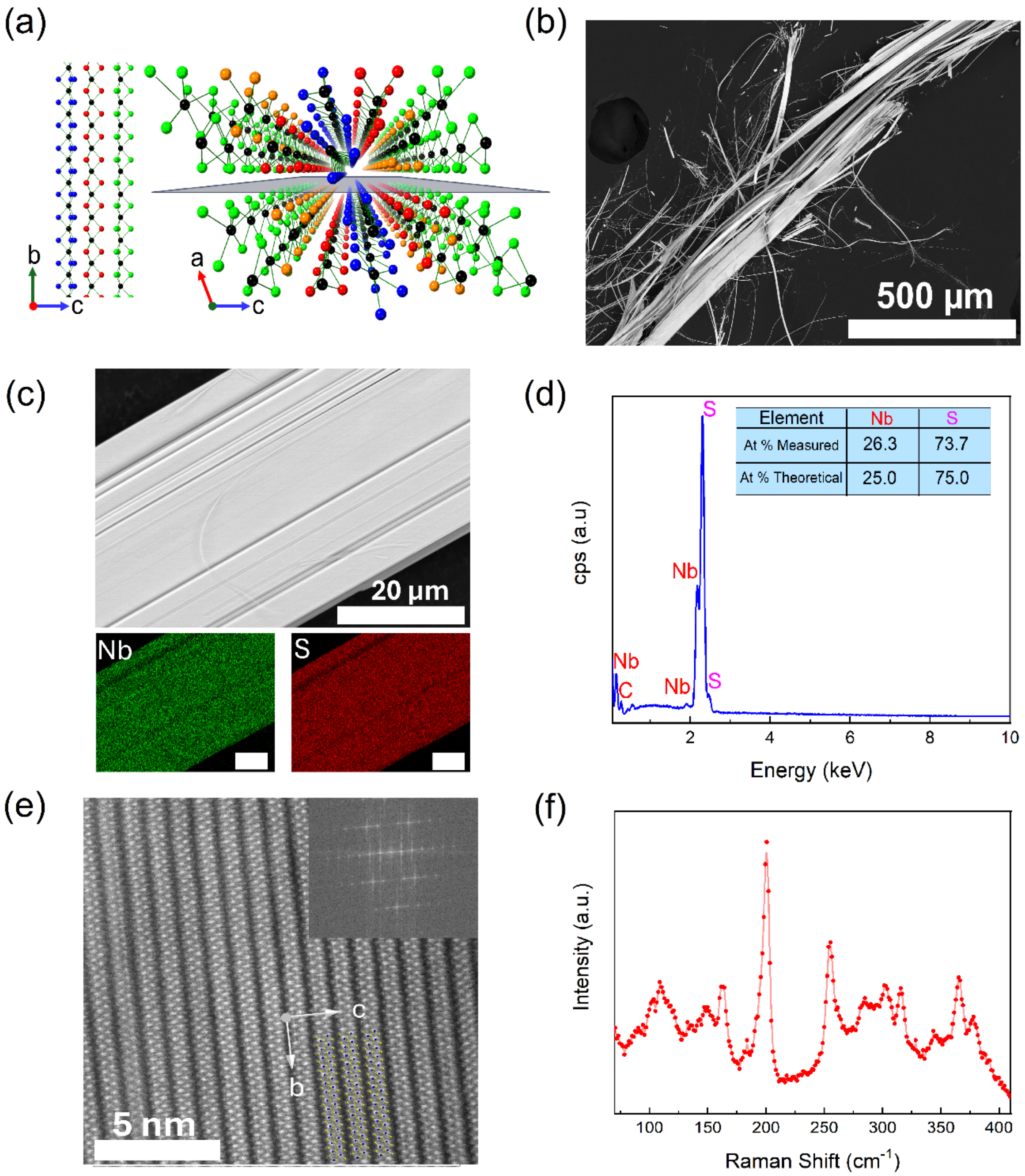

Supplemental Figure S11: **NbS_3 material characterizations data**. (a) Schematic of the NbS_3–II crystal structure with Nb atoms black, S atoms green, orange, blue, or red. Left: View down the *a*-axis, with unique NbS_3 chains color-coded. Right: Perspective view down the *b*-axis, where the gray plane indicates the preferred (100) cleavage. (b) SEM image of CVT-grown NbS_3 bulk single crystals. (c) SEM and EDS maps of the synthesized NbS_3 crystal. The elements Nb and S are represented in green and red colors, respectively. The scale bar is 10 µm. (d) EDS spectrum from an exfoliated NbS_3 crystal, with peaks labeled with their corresponding elements (carbon impurity comes from the carbon tape used for sample mounting). The inset table shows the atomic percentages of each element. (e) HRTEM image of an NbS_3 nanowire showing clear atomic chains (atomic structure overlay in lower right) and inset FFT. (f) Raman spectrum of NbS_3 nanowires.

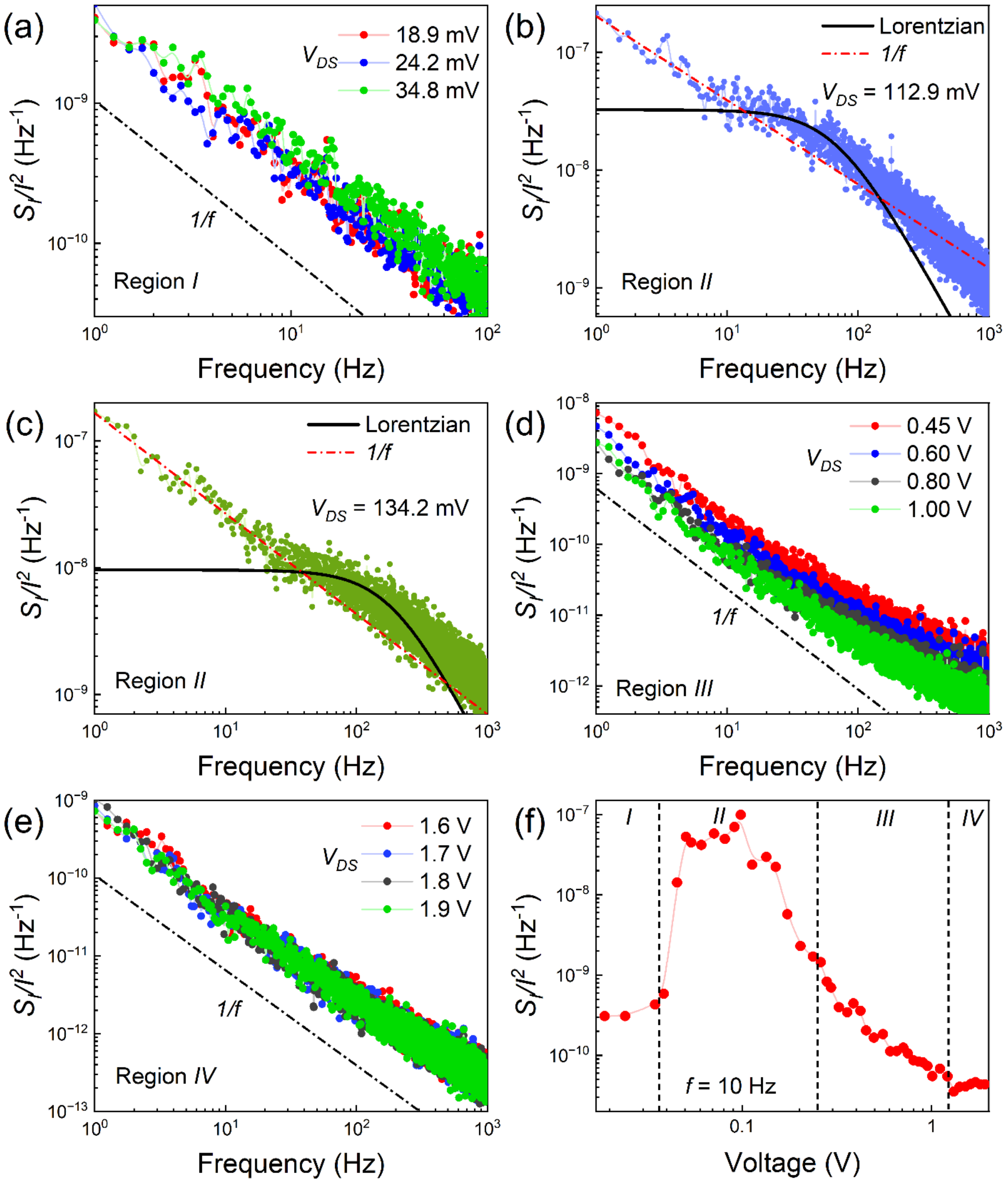

Supplemental Figure S12: **Noise data for a NbS_3-II nanowire device measured at T = 320 K**. The normalized noise spectral density, S_I/I^2, at (a) lower biases (region *I*), (b)-(c) across the depinning (region *II*), (d) in the CDW sliding region (region *III*), and (e) at higher bias saturation region (region *IV*). The noise is of *1/f* type, with some signatures of the Lorentzian bulges across depinning. The 60 Hz harmonics due to the power grid from the spectra were removed during data analysis. (f) The noise, S_I/I^2, at fixed frequency f =10 Hz *vs.* bias voltage, indicating the bias ranges of each measured region.

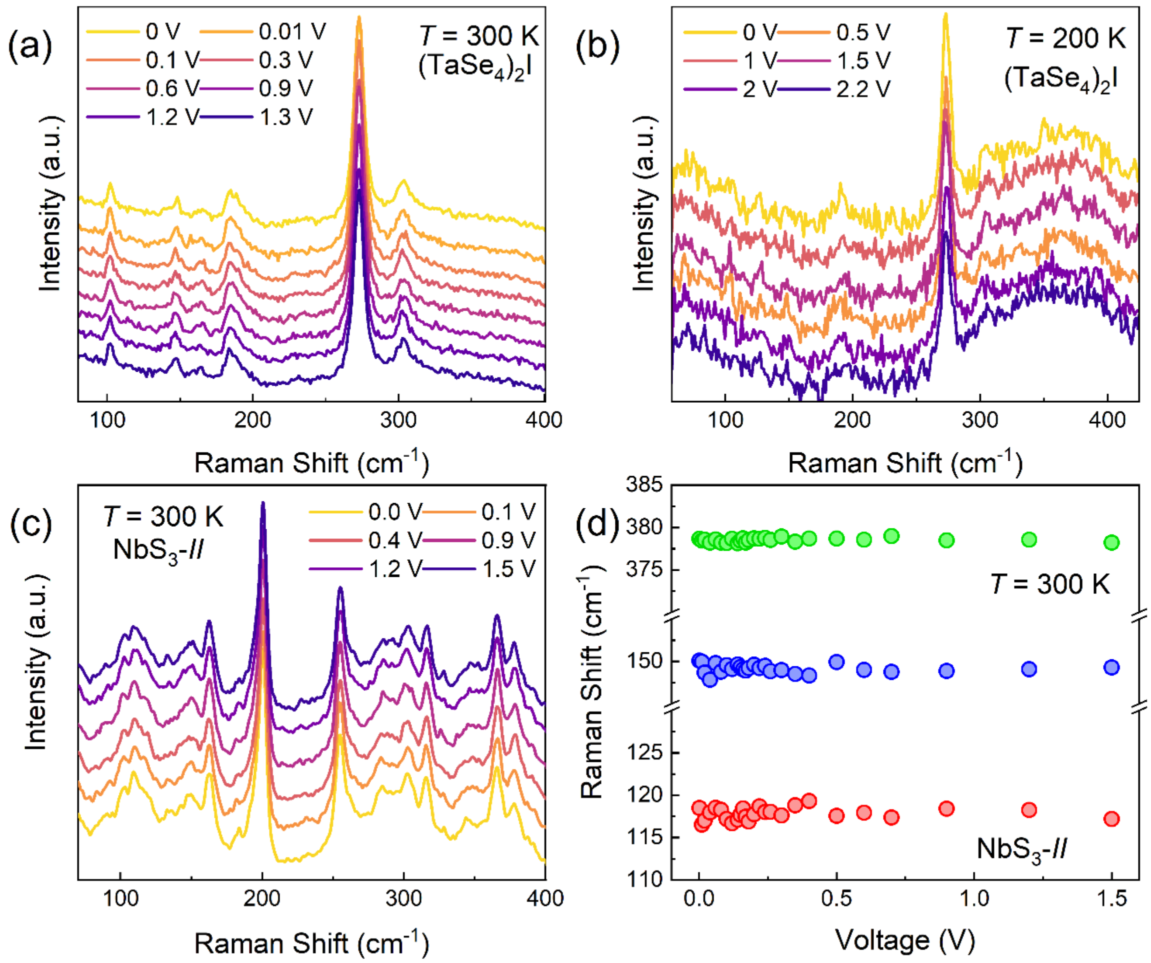

Supplemental Figure S13: **In-situ bias-dependent Raman spectroscopy**. The Raman spectra of $(TaSe_4)_2I$ nanowire device measured at different bias voltages at (a) $T = 300$ K, and (b) $T = 200$ K. The Raman peak positions remain nearly unchanged under applied bias, indicating no Joule heating effect. (c) The Raman spectra at different bias points measured on the NbS_3 nanowire device at $T = 300$ K. (d) The Raman frequency shift for several phonon peaks in the NbS_3 device as a function of applied voltage. The positions of the Raman peaks stay constant under applied biases, proving the absence of any local heating.

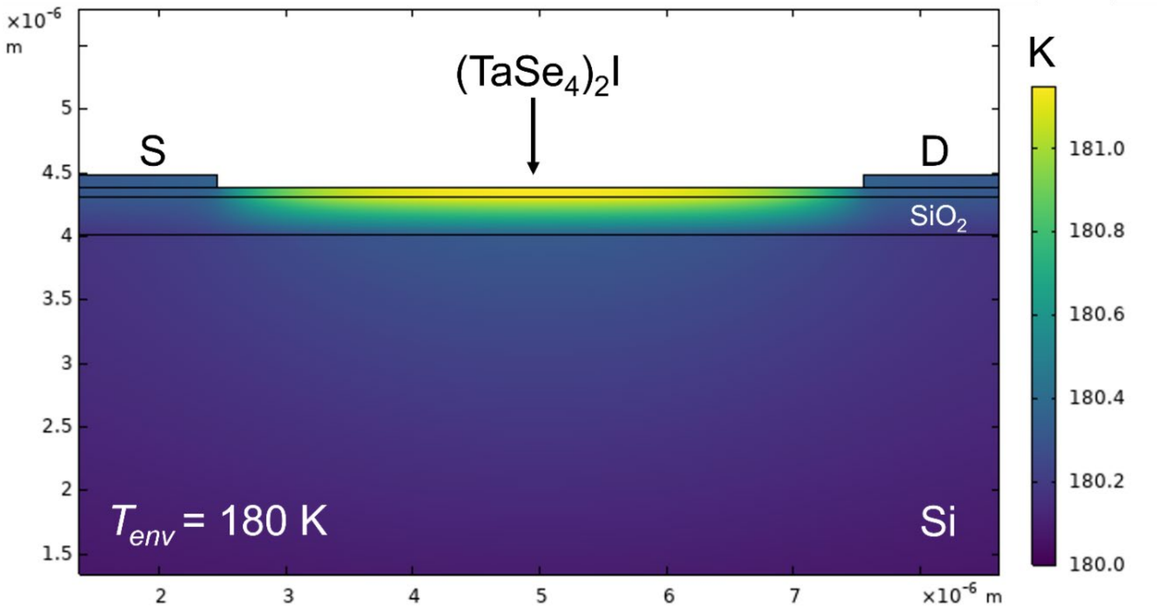

Supplemental Figure S14: Simulated temperature distribution on $(TaSe_4)_2I$ nanowire device on Si/SiO_2 substrate in two-terminal configuration at $T = 180$ K, measured at 0.9 V voltage drop. The finite element method is implemented in the COMSOL simulation. The temperature rise generated from Joule heating in the channel region is sensitive to the sample geometry, material properties, and ambient conditions. The simulated device geometry consists of an 83 nm thick $(TaSe_4)_2I$ layer with a 5.1 µm channel length, positioned on top of a SiO_2/Si substrate with thicknesses of 300 nm and 4 µm, respectively. The maximum temperature rise at the hot spot due to Joule heating at 0.9 V is ~ 1 K. The simulation data show that there were no local heating effects on measured noise characteristics.

I. The phenomenological theory of $1/f$ noise in non-linear approximation

We now derive the general phenomenological theory of $1/f$ noise in CDW conductors using a non-linear expression for current that captures transport characteristics of CDW conductors with low damping. The initial expression for the current can be chosen to match the specific material of interest closely. Here, we start with the equation for $(TaSe_4)_2I$, a Weyl CDW conductor with low damping. The total current can be best described by the collective current with two non-linear components:

$$I = I_n + I_c = I_n + \left(I_{c_1} + I_{c_2}\right) = G_n V + \Gamma_c \left[(V^2 - V_t^2)^a + (V - V_t)^{2a} \right] \theta(V - V_t). \tag{S1}$$

I_{c_1} and I_{c_2} represent two collective current components with different non-linear voltage dependencies.

Let us assume uncorrelated, zero-mean fluctuations for, $\delta G_n, \delta \Gamma_c, \delta V_t$. Then

$$\delta I = \frac{\partial I}{\partial G_n}\delta G_n + \frac{\partial I}{\partial \Gamma_c}\delta \Gamma_c + \frac{\partial I}{\partial V_t}\delta V_t \ , \tag{S2}$$

$$\frac{\partial I}{\partial G_n} = V, \tag{S3}$$

$$\frac{\partial I}{\partial \Gamma_c} = (V^2 - V_t^2)^a + (V - V_t)^{2a} \ , \tag{S4}$$

$$\frac{\partial I}{\partial V_t} = -2a\, \Gamma_c \left[V_t\, (V^2 - V_t^2)^{(a-1)} + (V - V_t)^{(2a-1)}\right]. \tag{S5}$$

We can write the current fluctuation as:

$$\delta I = V\delta G_n + [(V^2 - V_t^2)^a + (V - V_t)^{2a}]\delta\Gamma_c - 2a\, \Gamma_c \left[V_t\, (V^2 - V_t^2)^{(a-1)} + (V - V_t)^{(2a-1)}\right]\delta V_t \ , \tag{S6}$$

$$\delta I = \frac{G_n V \delta G_n}{G_n} + \frac{\Gamma_c\left[(V^2 - V_t^2)^a + (V - V_t)^{2a}\right]\delta\Gamma_c}{\Gamma_c} - 2a\, \Gamma_c \frac{V_t\left[V_t^2\, (V^2 - V_t^2)^{(a-1)} + V_t (V - V_t)^{(2a-1)}\right]\delta V_t}{V_t} \ , \tag{S7}$$

$$\delta I = \delta G_n \frac{V G_n}{G_n} + \delta\Gamma_c \frac{\Gamma_c\left[(V^2 - V_t^2)^a + (V - V_t)^{2a}\right]}{\Gamma_c} - \frac{2a\, \Gamma_c\left[V_t^2\, (V^2 - V_t^2)^{(a-1)} + V_t (V - V_t)^{(2a-1)}\right]\delta V_t}{V_t} \ , \tag{S8}$$

$$\delta I = \frac{\delta G_n}{G_n} I_n + \frac{\delta\Gamma_c}{\Gamma_c} I_c - \left[\delta V_t \times 2a\, \Gamma_c \frac{\left\{V_t^2\, (V^2 - V_t^2)^{(a-1)} + V_t (V - V_t)^{(2a-1)}\right\}}{V_t}\right]. \tag{S9}$$

Dividing by the total current, we obtain:

$$\frac{\delta I}{I} = \left(\frac{\delta G_n}{G_n}\right)\left(\frac{I_n}{I}\right) + \left(\frac{\delta \Gamma_c}{\Gamma_c}\right)\left(\frac{I_c}{I}\right) - \left(\frac{\delta V_t}{V_t}\right)\left[2a\Gamma_c \frac{\left\{V_t^2\left(V^2 - V_t^2\right)^{(a-1)} + V_t(V - V_t)^{(2a-1)}\right\}}{I}\right]. \quad \text{(S10)}$$

As the next step, we square, take the ensemble average, and assume no cross correlation to get to the final expression for noise in this non-linear model:

$$\frac{\langle \delta I^2 \rangle}{I^2} = \frac{\langle \delta G_n^2 \rangle}{G_n^2}\left(\frac{I_n}{I}\right)^2 + \frac{\langle \delta \Gamma_c^2 \rangle}{\Gamma_c^2}\left(\frac{I_c}{I}\right)^2 + \frac{\langle \delta V_t^2 \rangle}{V_t^2}\left[2a\,\Gamma_c \frac{\left\{V_t^2\left(V^2 - V_t^2\right)^{(a-1)} + V_t(V - V_t)^{(2a-1)}\right\}}{I}\right]^2, \quad \text{(S11)}$$

$$\frac{\langle \delta I^2 \rangle}{I^2} = \frac{\langle \delta G_n^2 \rangle}{G_n^2}\left(\frac{I_n}{I}\right)^2 + \frac{\langle \delta \Gamma_c^2 \rangle}{\Gamma_c^2}\left(\frac{I_c}{I}\right)^2 + \frac{\langle \delta V_t^2 \rangle}{V_t^2}\,4a^2\,\Gamma_c^2 \frac{\left[V_t^2\left(V^2 - V_t^2\right)^{(a-1)} + V_t(V - V_t)^{(2a-1)}\right]^2}{I^2}, \quad \text{(S12)}$$

$$\frac{\langle \delta I^2 \rangle}{I^2} = \frac{\langle \delta G_n^2 \rangle}{G_n^2}\left(\frac{I_n}{I}\right)^2 + \frac{\langle \delta \Gamma_c^2 \rangle}{\Gamma_c^2}\left(\frac{I_c}{I}\right)^2 + \frac{\langle \delta V_t^2 \rangle}{V_t^2} \times f(V, V_t), \quad \text{(S13)}$$

where,

$$f(V, V_t) = 4a^2\,\Gamma_c^2 \frac{\left[V_t^2\left(V^2 - V_t^2\right)^{(a-1)} + V_t(V - V_t)^{(2a-1)}\right]^2}{I^2}, \quad \text{(S14)}$$

$$f(V, V_t) = 4a^2\,\Gamma_c^2 \frac{\left[V_t^4\left(V^2 - V_t^2\right)^{2(a-1)} + V_t^2(V - V_t)^{2(2a-1)} + 2V_t^3\left(V^2 - V_t^2\right)^{(a-1)}(V - V_t)^{(2a-1)}\right]}{I^2}, \quad \text{(S15)}$$

$$f(V, V_t) = 4a^2\left[V_t^4 \frac{\Gamma_c^2\left(V^2 - V_t^2\right)^{2(a-1)}}{I^2} + V_t^2 \frac{\Gamma_c^2(V - V_t)^{2(2a-1)}}{I^2} + 2V_t^3 \frac{\Gamma_c^2\left(V^2 - V_t^2\right)^{2(a-1)}(V - V_t)^{2(2a-1)}}{I^2}\right], \quad \text{(S16)}$$

$$f(V, V_t) = 4a^2\left[V_t^4 \frac{\Gamma_c^2\left(V^2 - V_t^2\right)^{2a}}{\left(V^2 - V_t^2\right)^2 I^2} + V_t^2 \frac{\Gamma_c^2(V - V_t)^{4a}}{(V - V_t)^2 I^2} + 2V_t^3 \frac{\Gamma_c^2\left(V^2 - V_t^2\right)^{2a}(V - V_t)^{4a}}{\left(V^2 - V_t^2\right)(V - V_t) I^2}\right], \quad \text{(S17)}$$

$$f(V, V_t) = 4a^2\left[V_t^4 \frac{\left\{\Gamma_c\left(V^2 - V_t^2\right)^{a}\right\}^2}{\left(V^2 - V_t^2\right)^2 I^2} + V_t^2 \frac{\left\{\Gamma_c(V - V_t)^{2a}\right\}^2}{(V - V_t)^2 I^2} + 2V_t^3 \frac{\Gamma_c\left\{\left(V^2 - V_t^2\right)^{a} \times \Gamma_c(V - V_t)^{2a}\right\}^2}{\left(V^2 - V_t^2\right)(V - V_t) I^2}\right], \quad \text{(S18)}$$

$$f(V, V_t) = 4a^2\left[V_t^4 \frac{I_{c_1}^2}{\left(V^2 - V_t^2\right)^2 I^2} + V_t^2 \frac{I_{c_2}^2}{(V - V_t)^2 I^2} + 2V_t^3 \frac{I_{c_1} I_{c_2}}{\left(V^2 - V_t^2\right)(V - V_t) I^2}\right], \quad \text{(S19)}$$

$$f(V, V_t) = 4a^2\left[\frac{V_t^4}{\left(V^2 - V_t^2\right)^2}\frac{I_{c_1}^2}{I^2} + \frac{V_t^2}{(V - V_t)^2}\frac{I_{c_2}^2}{I^2} + \frac{2V_t^3}{\left(V^2 - V_t^2\right)(V - V_t)}\frac{I_{c_1} I_{c_2}}{I^2}\right]. \quad \text{(S20)}$$

By adding Eq. (S20) in Eq. (S13), the total noise fluctuation becomes,

$$\frac{\langle \delta I^2 \rangle}{I^2} = \frac{\langle \delta G_n^2 \rangle}{G_n^2}\left(\frac{I_n}{I}\right)^2 + \frac{\langle \delta \Gamma_c^2 \rangle}{\Gamma_c^2}\left(\frac{I_c}{I}\right)^2 + \frac{\langle \delta V_t^2 \rangle}{V_t^2}\, 4a^2 \left[\frac{V_t^4}{\left(V^2 - V_t^2\right)^2}\frac{I_{c_1}^2}{I^2} + \frac{V_t^2}{(V - V_t)^2}\frac{I_{c_2}^2}{I^2}\frac{2V_t^3}{(V^2 - V_t^2)(V - V_t)}\frac{I_{c_1} I_{c_2}}{I^2}\right]\theta(V - V_t)\,. \quad \text{(S21)}$$

This represents the noise model equation for a CDW device, where,

$$\frac{S_{t_1}}{I^2} = 4a^2 \frac{V_t^4}{\left(V^2 - V_t^2\right)^2}\frac{I_{c_1}^2}{I^2}\frac{\langle \delta V_t^2 \rangle}{V_t^2}\,, \quad \text{(S22)}$$

$$\frac{S_{t_2}}{I^2} = 4a^2 \frac{V_t^2}{(V - V_t)^2}\frac{I_{c_2}^2}{I^2}\frac{\langle \delta V_t^2 \rangle}{V_t^2}\,, \quad \text{(S23)}$$

$$\frac{S_{t_3}}{I^2} = 4a^2 \frac{2V_t^3}{(V^2 - V_t^2)(V - V_t)}\frac{I_{c_1} I_{c_2}}{I^2}\frac{\langle \delta V_t^2 \rangle}{V_t^2}\,. \quad \text{(S24)}$$

At $V = V_t^+$, the normalized noise due to fluctuations in V_t is singular for $a < 1$, but in the limit of large $V \gg V_t$, it falls off rapidly.

At $V \gg V_t$,

$\frac{S_{t_1}}{I^2}, \frac{S_{t_2}}{I^2}$ & $\frac{S_{t_3}}{I^2}$ falls off as $\frac{1}{V^4}, \frac{1}{V^2}$ & $\frac{1}{V^3}$ respectively, which corresponds to $\frac{1}{I^2}, \frac{1}{I}$ & $\frac{1}{I^{1.5}}$.

II. Fitting parameters from the I-V model

The I-V model equation is fitted with experimental data. The obtained fitting parameters are as follows:

Device	$G_n(S)$	Γ_c	$V_t\ (V)$	a
1	1.947×10^{-6}	6.72×10^{-6}	0.08241	0.9
2	3.912×10^{-6}	3.556×10^{-6}	0.04751	0.89393
3	1.638×10^{-6}	4.31×10^{-6}	0.04392	0.79445

III. **Fitting parameters for the noise model**

To fit the noise model equation with the experimental data, we plugged in values of the I-V fitted parameters (G_n, Γ_c, V_t & a). The noise amplitudes are not calculated from the first principles but rather determined by comparing the experimental data with the model. For that, we calculated the average constant noise level, S_I/I^2, of the normal carrier noise from the experimental data since $S_I/I^2 = constant$. The average noise is taken as the value of the linear noise amplitude, $\langle \delta {G_n}^2 \rangle / {G_n}^2$. The CDW noise amplitude, $\langle \delta {\Gamma_c}^2 \rangle / {\Gamma_c}^2$ cannot be determined accurately since the experimental noise never saturates. We took the upper-bound value before the noise could be saturated at higher biases. For the threshold field noise amplitude, $\langle \delta {V_t}^2 \rangle / {V_t}^2$, we plugged in different values and superimposed the noise model output with the experimental data. The threshold amplitude was determined based on the best fit of the model with the experimental data at higher biases, where the noise follows, $S_I/I^2 \propto 1/I$.

The table contains the values used for the fluctuation terms to fit the noise model.

Device	$\frac{\langle \delta {G_n}^2 \rangle}{{G_n}^2}$	$\frac{\langle \delta \Gamma_c^2 \rangle}{\Gamma_c^2}$	$\frac{\langle \delta V_t^2 \rangle}{V_t^2}$
1	4.9×10^{-9}	1.0×10^{-12}	3.5×10^{-7}
2	9.5×10^{-9}	1.0×10^{-12}	1.9×10^{-6}
3	1.3×10^{-9}	1.0×10^{-12}	1.45×10^{-6}

IV. **Calculation of the transport parameters and weighted noise contributions**

In the incommensurate phase, total device current combines linear current and CDW current, such that: $I = I_n + I_c$, where, I_n is normal current, I_c is the CDW current, G is the total conductance, which combines linear and CDW terms, such as, $G = G_n + G_c$. Here, G_n is normal carrier conduction, which is calculated as the slope of I-V data in the linear regime, such as, $G_n = \frac{dI_n}{dV}$ and $G_c = \frac{dI}{dV} - G_n$, is the CDW carrier conductance, where $G = \frac{dI}{dV}$. The CDW current is calculated as,

$I_c = I - I_n = I - VG_n$. We now derive Eq. (1) of the main text. In the two-fluid model, total device current is given by the equation: $I = VG = I_n + I_c$. If the fluctuations in the two conductivities are independent and the cross-correlation is neglected, we get the total current fluctuations as:

$$\delta I = \delta I_n + \delta I_c = V(\delta G_n + \delta G_c). \quad \text{(S25)}$$

Taking the square of the fluctuation terms and neglecting the cross-correlated term, we have:

$$\delta I^2 = V^2 \delta {G_n}^2 + V^2 \delta {G_c}^2 \ . \quad \text{(S26)}$$

Taking the ensemble average, we obtain:

$$\langle \delta I^2 \rangle = V^2 \langle \delta {G_n}^2 \rangle + V^2 \langle \delta {G_c}^2 \rangle \ . \quad \text{(S27)}$$

Writing in terms of spectral density for fluctuations, we get:

$$S_I = V^2 S_{G_n} + V^2 S_{G_c}. \quad \text{(S28)}$$

Dividing by the square of I, we write:

$$\frac{S_I}{I^2} = S_{G_n} \frac{V^2}{I^2} + S_{G_c} \frac{V^2}{I^2} \ , \quad \text{(S29)}$$

$$\frac{S_I}{I^2} = S_{G_n} \frac{1}{G^2} + S_{G_c} \frac{1}{G^2} \ . \quad \text{(S30)}$$

Therefore, the total current fluctuations, can be written as:

$$\frac{S_I}{I^2} = \frac{S_{G_n}}{{G_n}^2} \frac{{G_n}^2}{(G_n+G_c)^2} + \frac{S_{G_c}}{{G_c}^2} \frac{{G_c}^2}{(G_n+G_c)^2} \ . \quad \text{(S31)}$$

Here, $\frac{S_I}{I^2}$ is the total normalized current noise; $\frac{{G_n}^2}{(G_n+G_c)^2}$, $\frac{{G_c}^2}{(G_n+G_c)^2}$ are the normalized conductions; $\frac{S_{G_n}}{{G_n}^2}$, $\frac{S_{G_c}}{{G_c}^2}$ are the normal and the CDW carrier conduction noise components; $\frac{S_{G_n}}{{G_n}^2} \frac{{G_n}^2}{(G_n+G_c)^2}$, $\frac{S_{G_c}}{{G_c}^2} \frac{{G_c}^2}{(G_n+G_c)^2}$ are the weighted contributions of the normal and CDW carrier conduction noise components.
